# Intelligent Neutrosophic Diagnostic System for Cardiotocography Data

**DOI:** 10.1155/2021/6656770

**Published:** 2021-02-10

**Authors:** Belal Amin, A. A. Salama, I. M. El-Henawy, Khaled Mahfouz, Mona G. Gafar

**Affiliations:** ^1^Port Said University, Faculty of Sciences, Port Said, Egypt; ^2^Zagazig University, Faculty of Computers and Information, Zagazig, Egypt; ^3^Department of Computer Science, College of Science and Humanities in Al-Sulail, Prince Sattam bin Abdulaziz University, Kharj, Saudi Arabia; ^4^Department of Machine Learning and Information Retrieval, Faculty of Artificial Intelligence, Kafrelsheikh University, Kafrelsheikh, Egypt

## Abstract

Cardiotocography data uncertainty is a critical task for the classification in biomedical field. Constructing good and efficient classifier via machine learning algorithms is necessary to help doctors in diagnosing the state of fetus heart rate. The proposed neutrosophic diagnostic system is an Interval Neutrosophic Rough Neural Network framework based on the backpropagation algorithm. It benefits from the advantages of neutrosophic set theory not only to improve the performance of rough neural networks but also to achieve a better performance than the other algorithms. The experimental results visualize the data using the boxplot for better understanding of attribute distribution. The performance measurement of the confusion matrix for the proposed framework is 95.1, 94.95, 95.2, and 95.1 concerning accuracy rate, precision, recall, and *F*1-score, respectively. WEKA application is used to analyse cardiotocography data performance measurement of different algorithms, e.g., neural network, decision table, the nearest neighbor, and rough neural network. The comparison with other algorithms shows that the proposed framework is both feasible and efficient classifier. Additionally, the receiver operation characteristic curve displays the proposed framework classifications of the pathologic, normal, and suspicious states by 0.93, 0.90, and 0.85 areas that are considered high and acceptable under the curve, respectively. Improving the performance measurements of the proposed framework by removing ineffective attributes via feature selection would be suitable advancement in the future. Moreover, the proposed framework can also be used in various real-life problems such as classification of coronavirus, social media, and satellite image.

## 1. Introduction

Vulnerability is the focal, basic reality in the medical field. Patients' feelings, specialists' observations, and lab results cannot be exactly reported. Clinical scientists cannot accurately characterize how illnesses adjust the ordinary working of the body. Generally, uncertainty [1] is a serious challenge for decision-makers at any organization and especially in the medical field. Doctors need to handle fast and accurate decisions, which are critical to human health. Cardiotocography (CTG) [2, 3] is a significant medical device early monitoring fetus distress by gynecologist. It is a graphical recording for both fetus heart rate and uterine contraction at the same time. Hence, it is necessary to analyse and interpret the CTG recordings of fetus health. The CTG dataset is commonly used by machine learning and classification researchers [4]. Gynecologists are interested, in most states, in classifying “fetus well-being.” They used two healthy or pathologic classes, but they, practically, classify fetus state into three classes: normal, suspicious, and pathologic (NSP). The CTG dataset is composed of 21 input attributes and 3 output classes (normal, suspicious, and pathologic). The total of datasets includes 2126 instances, and it is publicly available at the data-mining repository of the University of California, Irvine (UCI) [5].

Neutrosophic set theory (NS) [6, 7] is an improved, mathematical model that deals with uncertain and ambiguous data. It was firstly presented in 1995 by Smarandache, as an extension of the fuzzy system [4–7]. Fuzzy classifiers deal with uncertain data by degrees of truth. Neutrosophic classifier defines three functions; they are as follows: true, false, and indeterminacy degrees of handling uncertainty. These functions take a ratio between 0 and 1. Handling uncertain data by neutrosophic technique gives it a more accurate description and reduces the degree of randomness in data leading to make the most optimal decisions. NS theory has applications [6] in many fields such as mathematics, computer science, medical, physics, and others.

Many data-mining researchers generally decided to face these challenges of uncertainty in medical data and the CTG dataset, especially via various algorithms and techniques such as classification, clustering, association, and regression [8, 9], to analyse large amounts of data and achieve high accuracy. This research proposes a neutrosophic diagnostic system for classifying the uncertain medical CTG data; the proposed model is an integration of multilayer RNN [10, 11] and the interval neutrosophic set (INS) [12] concepts. RNN is built on neural network (NN) structure [13, 14] and rough set (RS) [10, 11] theory. NN is characterized by various advantages such as fault tolerance, simple structure, the capability of parallel processing of both datasets, and self-adaption. RS has various advantages as it performs sustainable amount of uncertain data and reduction attributes without losing information whereas INS is an extension of the NS, and it depends on three functions of truth, indeterminacy, and falsity-memberships. They are expressed by interval values not the binary ones. Obviously, INS can conveniently describe complex information.

The proposed research is important as it provides an efficient framework for multiclassification of CTG data concerning the fetus heart rate. Moreover, comparing its results with different fuzzy algorithms and techniques is to ensure that it achieves a good performance measurement in accuracy rate, precision, recall, and *F*-measure [15, 16]. It also provides an analysis of CTG attributes using WEKA mining tool to visualize it. The implementation model and the performance measurement on the CTG dataset are shown in the experiments, which prove the feasibility of the proposed neutrosophic diagnostic system.

The rest of the study is organized as follows: Section 2 discusses literature review and preliminaries of both theories and techniques used in the proposed model.  Section 3 presents the proposed neutrosophic diagnostic system and its advantages.  Section 4 provides the experimental result of the system and an analysis for other classification techniques. Also, it presents the comparisons between different measurement performances. Finally, Section 5 is the conclusion and future work.

## 2. Related Work and Preliminaries

This section gives a quick review on most relevant studies done into the medical field and a theoretical background of RNN and INS used in building the neutrosophic diagnostic framework.

### 2.1. Related Work

Many data-mining scientists and researchers presented studies for dealing with the uncertainty and ambiguity of medical data. They aimed at analysing and classifying data in an efficient manner as well as achieving good performance measurements such as accuracy rate, precision, and recall. These studies help doctors in prioritizing critical cases that need quick intervention to save them, where they are at the back of their concerns due to self-diagnosis in comparison with other more stable cases, which are in their priority.

Sunder et al. [17] simulate a machine learning classification model for classifying CTG dataset using supervised artificial neural network (ANN) and support vector machine (SVM). Afterwards, they compared their performance with the unsupervised clustering techniques fuzzy *C*-means, *K*-means, and supervised SVM classification. The ANN classification model achieves a better performance than other classification and clustering techniques. Unfortunately, the implemented model did not classify the suspicious state as good performance as the other two states of normal and pathological fetus heart rate.

Kocamaz and Cömert [18] provide a comparison between various classification techniques of machine learning such as ANN [13, 14], SVM [19], extreme learning machine [20], radial basis function network [21], and random forest [22] in performance measurement using confusion matrix [23]. He proved that ANN is the most efficient in the recall and specificity measures. Nevertheless, he did not specify which algorithm is the best in general.

Joshi et al. [24] present two hybrid neurofuzzy schemes for classification and they cluster different real-world datasets to pattern recognition applications. They actually achieved a good statistical measure rather than the other traditional machine learning algorithms; however, they did not calculate accuracy rate in their algorithm.

Postorino and Versaci [25] designed neurofuzzy simulation of user-mode transportation with extensive roads that depend on estimating different rules and membership function to help travel users take optimal decisions. They also presented fuzzy curves and surfaces for this problem; the limitation in the model is not efficient with a large number of features. They did not compare their simulation results with other machine learning algorithms.

Cacciola et al. [26] provide a hybrid neurofuzzy model to predict hydrocarbons and other particular pollutant concentrations of air pollution in urban environments due to their danger on human health. The provided model has efficient tools as it holds the nonlinear universal approximation property. They improved the traditional neural network model by introducing fuzzy learning within the neuronal layers. However, they did not compare the statistical measurements of their model to other learning algorithms.

Mathur et al. [27] present an adaptive neurofuzzy inference strategy (ANFIS) to predict the in-socket residual limb temperature. It was simulated using MATLAB's Fuzzy Logic Toolbox and the GUI editor used to analyse its performance. The proposed strategy results show that the modelling technique has comparable performance metrics with the predictive ability with an accuracy of ±0.5°C and it is most efficient for noninvasive temperature monitoring. They did not measure other performances, e.g., precision, recall, and *F*1-score.

Das et al. [28] simulated a hybrid neurofuzzy and feature reduction (NF-FR) model to analyse data. The proposed NF-FR model uses a feature-based class pertinence fuzzification process for all patterns. They compare the proposed model NF-FR to other machine learning models ANN, NF, and ANNFR models. Various statistical performance measures such as accuracy rate, root-mean-square error, precision, recall, and *F*-measure prove a better performance for ten real-world datasets as well as the efficiency in eliminating redundant and noisy information with the least time of learning.

Price et al. [29] provide a new layer called the fuzzy layer into a structure of deep learning methods to investigate the powerful aggregation properties, which appear in fuzzy techniques. They added various advantages for fuzzy aggregation techniques such as flexibility and capability of implementation. On the other hand, it needs more improvement in the fuzzy layer for deep learning.

Ahmed Abou El-Fetouh et al. [10] present a rough neural network model (RNN) to classify and analyse the performance of breast cancer dataset depending on training data with different sizes. They compared the accuracy rate of the implemented model with neural network algorithm using the WEKA [30] tool to estimate its accuracy rate. However, they did not estimate the consumption time of the RNN [10, 11] model and they did not use more algorithms to be compared with the proposed model.

Gafar [31] proposes a diagnosing system of breast cancer using a hybrid of fuzzy rough feature selection and RNN. The fuzzy rough feature selection algorithm is used to find the best reduction, and the RNN is trained by the reduced dataset to learn the connection weights iteratively. The experimental comparisons show the proposed model accuracy and time complexities. Again, the research needed more comparisons with literature algorithms.

Amin et al. [32] provide an implementation of the RNN [10, 11] algorithm not only to classify CTG dataset but also to estimate each accuracy rate and time consumption of the proposed model. They used the WEKA tool to estimate the accuracy rates and time consumptions for various algorithms such as ANN [13, 14], decision table [33], bagging [34], the nearest neighbor [35], decision stump [36], and least square support vector machine algorithm [37]. And then, they compared the proposed model to these various algorithms; this comparison showed that the proposed model achieved the most efficient performance. Nevertheless, they did not estimate the other performance measurement of the RNN model such as precision and recall.

Kraipeerapun et al. [12] propose a model, which combines fuzzy neural networks and interval neutrosophic sets to classify uncertainty map cell data. The binary classes constitute deposits and barren based on input feature vectors representing exploration data. The model is limited to classify only two classes.

Kraipeerapun et al. [38] dealt with the limitations in the previous paper; they implemented a new model that combines neural networks and interval neutrosophic sets to have multiclassification. They presented an assessment of uncertainty classical datasets, e.g., balance, ecoli, glass, lenses, wine, yeast, and zoo from the UCI machine learning repository. Although they improved the performance of classification technique, they did not estimate confusion matrix and its measurements of performance.

### 2.2. Rough Neural Networks

Rough set theory [10, 11] is characterized by its capability to process sustainable amount of uncertain data, and it reduces the features of a dataset without losing its information. It classifies uncertain data space into two disjoint approximation sets (lower and upper); this classification is based on the values of the features of NN [13, 14, 17] that are distinguished by high capability on fault tolerance, simple structure, parallel processing of big data, and self-adapted. RNNs are a combination of rough set theory and NN to benefit from their advantages. RNNs [10, 11, 31] are inspired by the concepts of traditional NN in both their learning algorithm and structure of connections. The essential difference is the neuron, which is used in RNN formed from a pair of neurons. One neuron represents the upper approximation and the other represents the lower approximation of rough neuron. The overlap between upper and lower neurons helps them to exchange information.

RNN is a multilayered supervised machine learning technique; it is composed of one input layer, one or more hidden layers, and one output layer. Both input and output layers are formed from the traditional neurons. The input layer represents features of a dataset, while the output layer corresponds to the data classes. The hidden layers contain the rough core of the RNN, and they are formed from a number of rough neurons. Thus, these hidden layers of rough neurons are determined by the Baum–Haussler rule [39] in

(1) $$\begin{array}{c} N_{\text{hn}}=\frac{N_{\text{ts}}*Te}{N_{i}+N_{o}}, \end{array}$$

where *N*_hn_ is the number of hidden neurons; *N*_ts_ is the number of training samples, *Te* is the tolerance error, *N*_i_ is the number of inputs (attributes or features), and *N*_o_ is the number of the output.

RNN [10–32] applies feedforward algorithm where the values of CTG features are multiplied by randomly generated weights in both directions of upper and lower neurons using

(2) $$\begin{array}{c} I_{Ln}=\sum_{J=1}^{n}W_{Lnj}O_{nj},\\ I_{Un}=\sum_{J=1}^{n}W_{Unj}O_{nj}. \end{array}$$

The following equations compute the upper and lower neuron output by the maximum and minimum values of activation function, respectively:

(3) $$\begin{array}{c} O_{Ln}=\text{Min}\left(f\left(I_{Ln}\right),f\left(I_{Un}\right)\right),\\ O_{Un}=\text{Max}\left(f\left(I_{Ln}\right),f\left(I_{Un}\right)\right). \end{array}$$

The sigmoid function

(4) $$\begin{array}{c} f\left(x\right)=\frac{1}{1+e^{-\lambda x}}, \end{array}$$

where *λ* is a constant, is used as an activation function. The rough neuron output is computed by

(5) $$\begin{array}{c} O=O_{Ln}+O_{Un}. \end{array}$$

The actual class of RNN is compared with the target class of the CTG dataset. In case there is an error, the backpropagation algorithm computes the difference by

(6) $$\begin{array}{c} E=T-O. \end{array}$$

The backpropagation is applied to adjust the weights to get optimal ones. The upper and lower weights of the network are updated by

(7) $$\begin{array}{c} \text{new }w_{i}=\text{old }w_{i}+\gamma *\frac{\partial E}{\partial w_{i}}, \end{array}$$

where *γ* is the learning rate. The model repeats equations (2)–(7) until it reaches to the optimal weight RNN.

### 2.3. Interval Neutrosophic Set

Neutrosophic set theory [40] was introduced by Smarandache, as a generalization of other classical uncertainty theories such as fuzzy set theory, intuitionistic fuzzy set [4–7], an interval-valued intuitionistic fuzzy set [41, 42], dialetheist set, and paradoxist set [43]. Neutrosophic set continues to represent uncertainty, ambiguity, and incompleteness of data.

INS [12] is a paradigm of neutrosophic set where any element of the dataset is distinct by three values of true (*T*), indeterminacy (*I*), and false membership degrees (*F*), *T*, *I*, and *F* values ∈. [0, 1]. Hence, the general definition of INS is as follows: let *Z* is a space of instances of the dataset. INS in *Z* is defined as *P*={*Z* : (*T*_*p*_(*Z*); *I*_*p*_(*Z*); *F*_*p*_(*Z*))*|z* ∈ *Z*^∧^

(8) $$\begin{array}{c} T_{p}:X⟶{\left[0,1\right]}^{\wedge },\\ I_{p}:X⟶{\left[0,1\right]}^{\wedge },\\ F_{p}:X⟶{\left[0,1\right]}^{\wedge },\\ 0\leq T_{p}\left(z\right)+I_{p}\left(z\right)+F_{p}\left(z\right)\leq 3. \end{array}$$

The three memberships' values are the most independent cases.

## 3. The Proposed Interval Neutrosophic RNN Framework for Classifying CTG Data

IN-RNN is a proposed framework combining the rough concepts represented by RNN and indeterminacy concepts of interval neutrosophic set to handle uncertainty in CTG data. The IN-RNN provides a feasible postprocessing for uncertainty in predicting values of the RNN model using neutrosophic concepts for multiple classes. The framework is used to determine the state of fetal heart rate and other performance measurements, e.g., precision, recall, and f-score.

**Figure 1:**
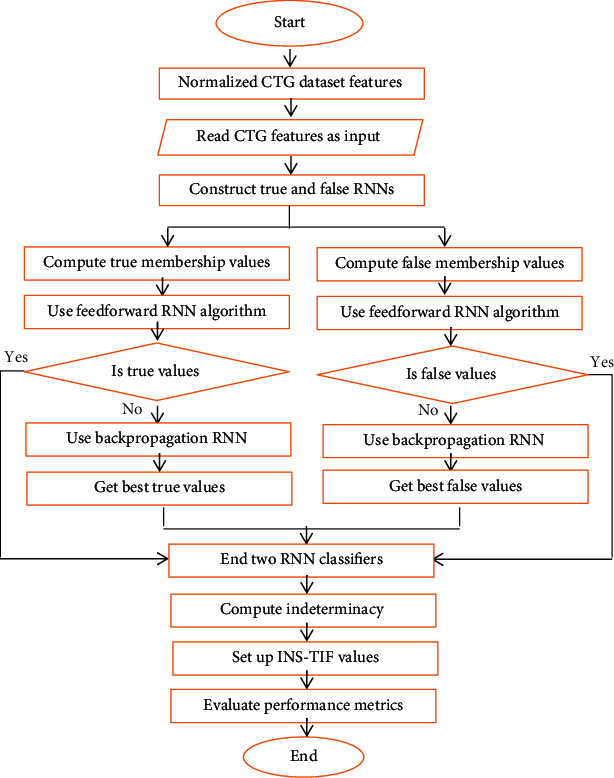
Flowchart of the proposed IN-RNN framework steps for classifying the CTG dataset.

IN-RNNs are built on two independent feedforward backpropagation RNNs with the same architecture and behavior to predict scaling values of output classes; also, they are trained by the same attributes as input vectors. The first RNN predicts true membership values (*T*), and the other predicts false membership values (*F*). The results of both networks produce uncertainty boundary zone to calculate indeterminacy values (*I*), (TIF) values form interval neutrosophic set (INS), so the final decision of such classification is characterized by INS-TIF values, as illustrated in Figure 1.

**Figure 2:**
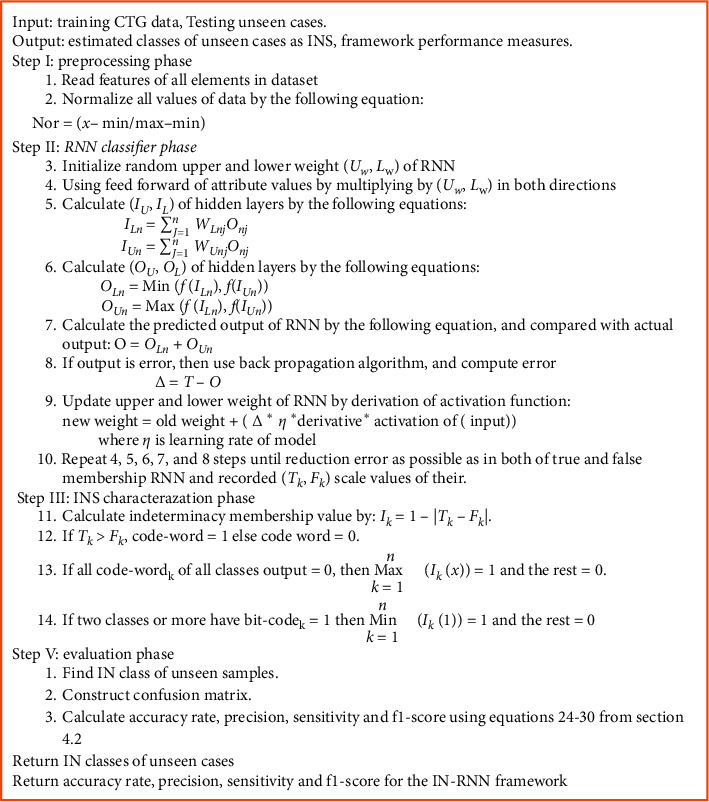
Description of the main phases of the IN-RNN framework algorithm.

The main difference between the two RNNs is the false RNN train for predicting the complement target value (code-word) of true RNNs. The code-word length equals the number of output classes. For instance, provided the code-word of *k*-th class has a value as 1 at the *k*^th^ bit and the rest is equal to 0 in training true RNNs, the code-word of *k*^th^ class at *k*^th^ bit in false RNNs will have a value 0 and the rest equals 1. In the IN-RNN model, the binary prediction of multiple classifications depends significantly on the true membership code-word by the equation in Step III (12) in the algorithm presented in Figure 2. In cases of inconsistency where code-word is 0 or more than one bit that equals 1 in the same code-word, equations in Step III (13-14) are used to make the final decision.

The predicted values of true and false membership RNN are nearly opposite to each other, in case the predicted value of true RNN is high and then the predicted value of false membership RNN should be low. Consequently, the uncertainty boundary zone appears from the inconsistency of them. Based on the INS definition, in Section 2.3, equations (8), indeterminacy membership value can be estimated from the difference between true and false membership values. The uncertainty is high if the difference between them is low and vice versa.

The proposed IN-RNN framework is established from four main phases: preprocessing, RNN classifier, INS characterization, and performance evaluation phases. Figure 2 illustrates the sequence of the IN-RNN phases in algorithmic shape.

Preprocessing phase: the medical data are normalized not only to preprocess irregularity in the attributed values but also to improve the performance of RNN in the implemented state.
RNN classifier phase: the RNN model is trained to get the best weights on true and false membership RNNs to estimate scale values of their networks by using the backpropagation algorithm. The normalized input data are multiplied by its weight and computed in the sigmoid activation function:
  (9) $$\begin{array}{c} f\left(x\right)=\frac{1}{1+e^{-\lambda x}}. \end{array}$$
INS characterization phase: indeterminacy membership degree is calculated using true and false membership values to form an interval neutrosophic set (INS). This phase makes the framework more informative by setting the indeterminacy of instances' classes to improve the performance of multiclassification of the CTG dataset.
Performance evaluation phase: the IN-RNN framework is tested using unseen cases to calculate performance measurements such as accuracy rate, precision, sensitivity, and *F*1-score. The efficiency of IN-RNNs is determined with respect to other classification algorithms.

## 4. Experimental Results

### 4.1. Dataset Visualization and Boxplot

**Figure 3:**
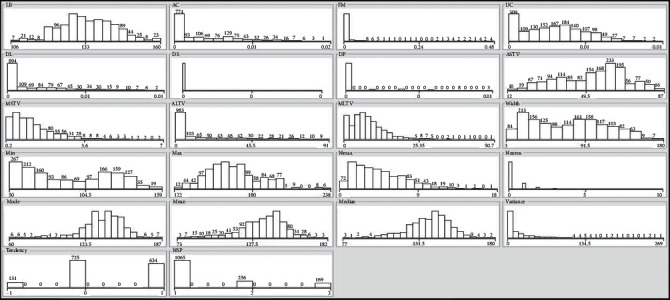
Visualization of CTG attributes using WEKA application tools.

The cardiotocography (CTG) dataset is used to train and test the IN-RNN framework and other machine learning algorithms, in the literature during the comparative study. The CTG dataset is downloaded from the website of the University of California, Irvine (UCI), machine learning repository. CTG has 2126 instances, and 21 inputs attribute to determine the state of fetal heart rate and uterine contraction at the same time. Depending on these attribute values, gynecologists could classify the state of fetal as normal, pathologic, or suspicious state (NSP) class. Therefore, it is critical to visualize [44] CTG attributes by using WEKA version 3.8.4 [30–45] tools, as in Figure 3. The attribute is drawn to illustrate a visual qualitative understanding of the distribution.

**Figure 4:**
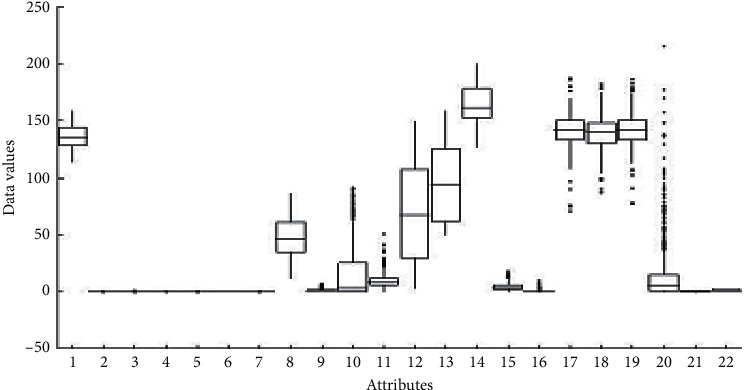
Boxplot of CTG attributes.

A boxplot [46] is a graphical statistical manner to summarize large amounts of data per each attribute and display five important statistic measurements such as minimum, maximum, median, range, and distribution of data. Likewise, it displays data symmetry as well as the upper and lower quartiles, which represent the numbers above and below the high and lower quarters of data. A boxplot of the CTG dataset is shown in Figure 4.

### 4.2. Experimental Setup and Results

The proposed IN-RNN framework for multiclassification is simulated by python 3.8 programming language on 64-bit operating Windows machine, processor Intel ®core™ i5 and RAM 4 GB.

Standard performance measures (e.g., accuracy rate, precision, recall (sensitivity), and *F*1-score) [4–38] derived from the confusion matrix [23] can be utilized for measuring IN-RNN framework efficiency.

#### 4.2.1. Confusion Matrix

Confusion matrix, Table 1, is a simple matrix for visualizing the multiclassification results of data related to predicted classes (*P*) and actual classes (*A*).

From the confusion matrix, the performance of a classifier can be estimated based on four important outcomes:

1. True positive (TP): if both predicted and actual classes are true.
2. True negative (TN): if both predicted and actual classes are false
3. False positive (FP): if predicted class is true and actual class is false.
4. False negative (FN): if predicted class is false and actual class is true.

#### 4.2.2. Accuracy Rate

The accuracy rate is a very traditional measure in evaluating the efficiency of a classifier; the general formula for estimating the accuracy rate is

(10) $$\begin{array}{c} \text{accuracy}=\frac{\sum_{i=1}^{n}\left(\text{TP}i+\text{TN}i\right)/\left(\text{TP}i+\text{TN}i+\text{FP}i+\text{FN}i\right)}{k}, \end{array}$$

where *k* is number of data samples.

#### 4.2.3. Precision

Precision is interested in the positive prediction of each individual class only. It can be estimated on the whole testing data as a weighted averaged:

(11) $$\begin{array}{c} \text{precision}\left(i\right)=\frac{\text{TP}i}{\text{TP}i+\text{FP}i}, \end{array}$$

where *i* = 1,2,3,….., *n* class,

(12) $$\begin{array}{c} {\text{precision}}_{\text{weighted average}}=\frac{\sum_{i=1}^{m}yi\left(\text{TP}I|/|\left(\text{TP}i+\text{FP}i\right)\right)}{\sum_{i=1}^{m}yi}. \end{array}$$

#### 4.2.4. Recall

Recall is the ratio between true positive prediction observations to the total observations with respect to each actual class individually. It can be estimated on the whole testing data as a weighted average:

(13) $$\begin{array}{c} \text{recall}\left(i\right)=\frac{\text{TP}i}{\text{TP}i+\text{FN}i}, \end{array}$$

(14) $$\begin{array}{c} {\text{recall}}_{\text{weighted average}}=\frac{\sum_{i=1}^{n}yi\left(\text{TP}I|/|\left(\text{TP}i+\text{FN}i\right)\right)}{\sum_{i=1}^{n}yi}. \end{array}$$

#### 4.2.5. *F*1-Score Measurement

*F*1-score depends on precision and recall measurement, and it considers the weighted average of them. Therefore, false positive and false negative samples are used in the evaluation. Intuitively, it is not as easy to understand it as accuracy. *F*1-score is useful more than accuracy rate in a state of disparate distribution of classification. It can be evaluated individually for each class and for the whole testing data by the following equations:

(15) $$\begin{array}{c} F1-\text{score}\left(i\right)=\frac{2x\text{precision}ix\text{sensitivity}i}{\text{precision}i+\text{sensitivity}i}, \end{array}$$

(16) $$\begin{array}{c} F1-{\text{score}}_{\text{weighted average}}=\frac{2\text{xprecisionweighted Xsensitivityweighted}}{\text{precisionweighted}+\text{sensitivityweighted}}. \end{array}$$

### 4.3. Result Analysis

The performance of the IN-RNN framework was estimated in terms of overall classification performance measurements using the 5-fold cross-validation (CV) method [4–18]. The CTG dataset is randomly distributed into five equal size subsets while keeping the proportion of data from NSP classes in each fold is approximately the same as the whole dataset. Four subsets of data are used for training while the fifth subset is mutually used for testing. At the end, the result of these five-folds is considered the average of accuracy on whole folds.

The IN-RNN framework processes CTG data in four phases. While preprocessing, CTG data attributes are normalized to avoid irregularity of values. In RNN classifier phase, true and false RNNs are established. The networks learn their weights using the backpropagation algorithm. After achieving the best predicted scaled weights, the network's output is passed to the next phase. The INS characterization process estimates the indeterminacy values of the predicted classes depending on the INS definition to make decisions more informative in the uncertainty boundary zone. From *T*, *F*, and *I* membership values, the final predicted classification of dataset samples is measured as illustrated in Tables 2–6. In these tables, the CTG classes are coded as *N*, *S*, and *P* that are normal, suspicious, and pathologic, respectively.

Table 2 shows the scaled values of *T*, *I*, and *F* membership. *T* and *F* membership values are estimated via two RNNs and *I* membership values for the three classes as in the equation in Step III (11). Binary classification of each class of the CTG dataset can be determined using the equation in Step III (12) based on code-words as shown in Tables 3–5.  Table 6 shows the results of binary classification for the new instances; in the same table, three code-words are equal to “000” in the first and third instances. According to the equation in Step III (13), the maximum indeterminacy membership code-word should be equal to 1 and the rest equals to 0. From INS values for all classes in Table 2, the final decision of classification for the first and third instances can be determined as normal (*N*) and suspicious (*S*), respectively.

The final process evaluates the framework performance by testing unseen cases. Here, the performance of the IN-RNN framework is measured, e.g., accuracy rate, precision, recall, and *F*1-score depending on the confusion matrix.

The confusion matrix is constructed to analyse the overall performance of the proposed model by presenting the classification report, and Table 7 presents the number of correctly and incorrectly classified instances from the CTG data.

According to the comparisons with literature algorithms, WEKA application [20–40] is used to analyse the CTG dataset using different machine learning models such as nearest neighbor [23], neural network [12, 13], and decision table [23]. The estimated performance metrics for these models are shown in Table 8. The performance metric values show that the proposed model IN-RNNs achieves a better and more efficient performance than the other machine learning models.

**Figure 5:**
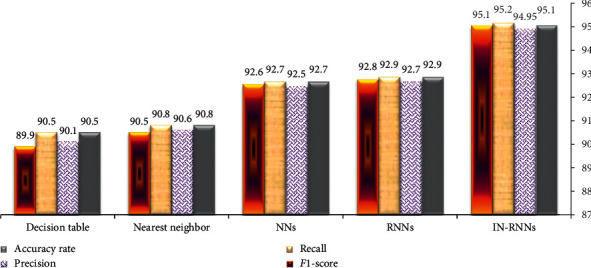
Comparison chart between the IN-RNN framework and fuzzy machine learning models in performance metrics.

Figure 5 represents a comparative chart between the IN-RNN framework and different machine learning models in their performance metrics.

Receiver operation characteristic (ROC) [6–33] is a pictorial tool for analysing the performance of the multiclassification models and estimates the area under the curve (AUC) for each class individually. AUC combines measures of recall (true positive) and specificity (true negative). In case AUC = 1 or approximate to 1, the classification model test is perfect without errors.

**Figure 6:**
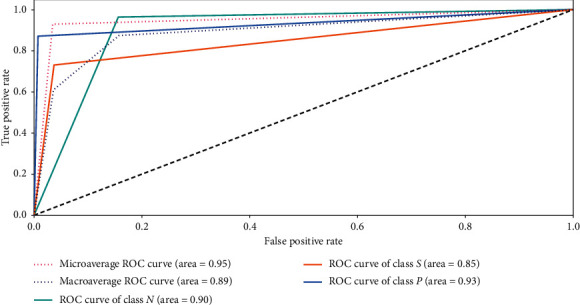
ROC analysis performance measurement of the IN-RNNs framework.

In the IN-RNN model, the ROC tool displays that the classification of CTG data is efficient for three classes, where it classifies pathologic, normal, and suspicious states by high AUC 0.93, 0.90, and 0.85 respectively, as shown in Figure 6.

## 5. Conclusion and Future Work

Uncertainty boundary zone in classifying CTG data is a vital issue. Neutrosophic theory is interested in estimating the uncertainty boundaries of data based on membership, truth, and indeterminacy values. Moreover, rough neural networks prove their ability to find uncertainty boundaries of the uncertain classes. While the proposed IN-RNNs consist of a hybrid framework between neutrosophic and rough theories, IN-RNN classifies multiple class CTG data in terms of neutrosophic set. The architecture of IN-RNNs is built via two independent backpropagation RNNs for evaluating the true and false memberships' values. The inconsistency between true and false values forms an indeterminacy membership value, while the three memberships form the interval neutrosophic decision class.

The experimental results present a distribution and boxplot visualization of CTG attributes by WEKA application. Concerning the performance evaluation, a cross validation is used to estimate the performance measurement of the IN-RNN framework with confusion matrix while ROC is employed in unseen cases of CTG data. In addition, different metrics, e.g., accuracy rate, precision, recall, and *F*1-score, are used to determine the efficiency of IN-RNN. WEKA application is employed to estimate performance metrics of several algorithms such as neural network, nearest neighbor, and decision table algorithm. The comparison between the IN-RNN model and other different machine learning models proves that the proposed model achieved more efficient and feasible performance in classifying CTG data.

In the future work, feature selection methods would be applied to remove ineffective attributes for improving performance measurement of the proposed model.

## Figures and Tables

**Table 1:** Confusion matrix form.

Actual	*A*_n…._.*A*_j……_*A*_1_	Predicted
		*P*_1_ … …*P*_*i*_ … …*P*_*n*_
		*x*_11_…….*x*_1*j*_……..*x*_1*n*_
		*x*_*i*1_……..*x*_*ij*_………*x*_*in*_
		*x*_*n*1_…….*x*_*nj*_………*x*_*nn*_

where *x*_*ij*_ represents the number of samples belonging to class *P*_*i*_ but predicted as class *A*_j_.

**Table 2:** The interval neutrosophic set (INS).

True membership values			Indeterminacy membership values			False membership values		
*N*_*t*_	*S*_*t*_	*P*_*t*_	*N*_*i*_	*S*_*i*_	*P*_*i*_	*N*_*f*_	*S*_*f*_	*P*_*f*_
0.50031	0.22042	0.02296	0.91964	0.33285	0.08015	0.58067	0.88756	0.94281
0.99950	0.00634	0.00000	0.08838	0.01891	0.02225	0.08788	0.98743	0.97775
0.19951	0.60074	0.12381	0.22239	0.72470	0.69965	0.97712	0.87604	0.42416
0.13034	0.57034	0.19766	0.23205	0.81184	0.25765	0.89829	0.38218	0.94000
0.14003	0.00432	0.98561	0.64750	0.00455	0.18190	0.49253	0.99977	0.16750

**Table 3:** Binary-code of *N*-class.

*N*_*t*_	*N*_*i*_	*N*_*f*_	Binary *N*-class
0.50031	0.91964	0.58067	0
0.99950	0.08838	0.08788	1
0.19951	0.22239	0.97712	0
0.13034	0.23205	0.89829	0
0.14003	0.64750	0.49253	0

**Table 4:** Binary-code of *S*-class.

*S*_*t*_	*S*_*i*_	*S*_*f*_	Binary *S*-class
0.22042	0.33285	0.88756	0
0.00634	0.01891	0.98743	0
0.60074	0.72470	0.87604	0
0.57034	0.81184	0.38218	1
0.00432	0.00455	0.99977	0

**Table 5:** Binary-code of *P*-class.

*P*_*t*_	*P*_*i*_	*P*_*f*_	Binary *P*-class
0.02296	0.08015	0.94281	0
0.00000	0.02225	0.97775	0
0.12381	0.69965	0.42416	0
0.19766	0.25765	0.94000	0
0.98561	0.18190	0.16750	1

**Table 6:** Neutrosophic classification of NSP class.

Binary N-class	Binary *S*-class	Binary *P*-class	INS of *N*-class	INS of *S*-class	INS of *P*-class	Predicted class	Actual class	State
0	0	0	1	0	0	N	N	True
1	0	0	1	0	0	N	N	True
0	0	0	0	1	0	S	S	True
0	1	0	0	1	0	S	S	True
0	0	1	0	0	1	P	P	True

**Table 7:** Confusion matrix of the IN-RNN model.

Predicted classes				
		Normal	Suspicious	Pathologic	Total
Actual classes	Normal	335	6	1	342
	Suspicious	11	41	0	52
	Pathologic	0	3	28	31
	Total	346	50	29	425

**Table 8:** Comparison between different machine learning models in performance metrics in (%).

Models	Accuracy rate	Precision	Recall	*F*1-score
IN-RNNs	95.1	94.95	95.2	95.1
RNNs	92.9	91.2	91.4	91.3
NNs	92.7	92.5	92.7	92.6
Nearest neighbor	90.8	90.6	90.8	90.5
Decision table	90.5	90.1	90.5	89.9

## Data Availability

The cardiotocography data in experiments are on the website with the following link http://archive.ics.uci.edu/ml/datasets/cardiotocography. (Accessed February 2019).
